# Metagenomics of Wastewater Influent from Southern California Wastewater Treatment Facilities in the Era of COVID-19

**DOI:** 10.1128/MRA.00907-20

**Published:** 2020-10-08

**Authors:** Jason A. Rothman, Theresa B. Loveless, Madison L. Griffith, Joshua A. Steele, John F. Griffith, Katrine L. Whiteson

**Affiliations:** a Department of Molecular Biology and Biochemistry, University of California, Irvine, Irvine, California, USA; b Department of Biomedical Engineering, University of California, Irvine, Irvine, California, USA; c Southern California Coastal Water Research Project, Costa Mesa, California, USA; DOE Joint Genome Institute

## Abstract

Sequencing wastewater may be useful for detecting pathogens and assaying microbial water quality. We concentrated, extracted, and sequenced nucleic acids from 17 composite influent wastewater samples spanning seven southern California wastewater treatment facilities in May 2020. Bacteria were the most proportionally abundant taxonomic group present, followed by viruses and archaea.

## ANNOUNCEMENT

Monitoring sewage provides population-level data on the diversity and spread of pathogens alongside clinical testing to assist in disease outbreak response (1–4). This approach may be able to determine the disease burden of a population, even when asymptomatic individuals are present (5). Likewise, the metagenomic sequencing of wastewater may also be useful in monitoring microbial water quality (6, 7) and the simultaneous detection of multiple pathogens.

Seventeen 24-hour composite wastewater influent samples were collected from seven locations across southern California during May 2020. In San Diego County, we collected samples from the South Bay Water Reclamation Plant (WRP), Hale Avenue Resource Recovery Facility, North City WRP, and Point Loma Wastewater Treatment Plant. In Los Angeles County, we collected samples from the San Jose Creek WRP, Hyperion WRP, and Joint Water Pollution Control Plant. We stored samples at 4°C for 0 to 40 days until extraction (Table 1).

**TABLE 1:** Descriptions of each sequenced sample^*a*^

Sample name	Wastewater facility	SRA accession no.	Collection date	Days stored at 4°C before extraction	Latitude/longitude
Joint Water PCP 5.28.20-13	Joint Water PCP	SRR12352293	28 May 2020	13	33.801023 N, 118.284708 W
Hyperion 5.27.20-14	Hyperion WRP	SRR12352294	27 May 2020	14	33.925506 N, 118.430709 W
South Bay 5.28.20-13	South Bay WRP	SRR12352295	28 May 2020	13	32.543403 N, 117.067960 W
San Jose Creek 5.28.20-1	San Jose Creek WRP	SRR12352296	28 May 2020	1	34.033844 N, 118.023501 W
Joint Water PCP 5.28.20-1	Joint Water PCP	SRR12352297	28 May 2020	1	33.801023 N, 118.284708 W
Hyperion 5.28.20-1	Hyperion WRP	SRR12352298	28 May 2020	1	33.925506 N, 118.430709 W
Point Loma 5.29.20-0	Point Loma WTP	SRR12352299	29 May 2020	0	32.679592 N, 117.246719 W
Hyperion 5.28.20-13	Hyperion WRP	SRR12352300	28 May 2020	13	33.925506 N, 118.430709 W
North City 5.29.20-0	North City WRP	SRR12352301	29 May 2020	0	32.878986 N, 117.198984 W
North City 5.29.20-12	North City WRP	SRR12352302	29 May 2020	12	32.878986 N, 117.198984 W
Point Loma 5.27.20-14	Point Loma WTP	SRR12352303	27 May 2020	14	32.679592 N, 117.246719 W
South Bay 5.28.20-1	South Bay WRP	SRR12352304	28 May 2020	1	32.543403 N, 117.067960 W
Hale Ave 5.5.20-36	Hale Avenue RRF	SRR12352305	5 May 2020	36	33.105224 N, 117.113170 W
Hale Ave 5.1.20-40	Hale Avenue RRF	SRR12352306	1 May 2020	40	33.105224 N, 117.113170 W
Hale Ave 5.4.20-37	Hale Avenue RRF	SRR12352307	4 May 2020	37	33.105224 N, 117.113170 W
San Jose Creek 5.29.20-12	San Jose Creek WRP	SRR12352308	29 May 2020	12	34.033844 N, 118.023501 W
Point Loma 5.28.20-13	Point Loma WTP	SRR12352309	28 May 2020	13	32.679592 N, 117.246719 W

a PCP, pollution control plant; WRP, water reclamation plant; WTP, water treatment plant; RRF, resource reclamation facility.

We followed a viral nucleic acid concentration and extraction protocol based on Wu et al. (8). We pasteurized 42.5 ml of wastewater at 60°C for 90 min and then filtered samples with a 0.22-μm filter (Corning, Corning, NY) into a tube containing 4.2 g polyethylene glycol 8000 (PEG-8000) and 0.95 g NaCl (Thermo Fisher, Waltham, MA). We then centrifuged the filtrate at 12,000 × *g* for 2 h at room temperature, aspirated off the supernatant, and centrifuged it again for 10 min. We removed the remaining supernatant and added 1.5 ml TRIzol (Thermo Fisher, Waltham, MA) to extract the nucleic acids, transferred the mixture to a 2-ml tube, and incubated it for 5 min at room temperature. We added 300 μl chloroform, vortexed the mixture for 1 min, incubated it for 5 min at room temperature, and then centrifuged it at 12,000 × *g* for 15 min at 4°C. We moved the aqueous phase to a new tube and added 1 volume of 4°C isopropanol and then incubated it for 15 min at room temperature, centrifuged it at 12,000 × *g* for 10 min at 4°C, and then removed the supernatant. We washed the pellets with cold 75% ethanol and then centrifuged them at 12,000 × *g* for 3 min at 4°C, followed by another wash. We air-dried the RNA pellets for 10 min and then resuspended them in 30 μl of RNase-free water and did not treat the RNA with DNase.

We generated cDNAs with SuperScript IV reverse transcriptase (Thermo Fisher, Waltham, MA) using the manufacturer’s protocol on 10.5 μl template RNA. All reaction mixtures included 40 units of recombinant human placenta RNase inhibitor (New England BioLabs, Ipswich, MA). We shipped cDNAs to the Microbial Genome Sequencing Center (MiGS, Pittsburgh, PA), which handled library preparation and sequencing. MiGS prepared paired-end libraries with the Flex for Enrichment kit (Illumina, San Diego, CA) and then sequenced the libraries as 2 × 150-bp paired-end reads on an Illumina NextSeq 550 instrument. We used bbduk (9) with default parameters for quality trimming and to remove artifacts and adapters. We assigned taxonomy to reads with Kraken 2 (10), removed human contamination, and visualized the data with ggplot2 (11) in R (12).

**FIG 1:**
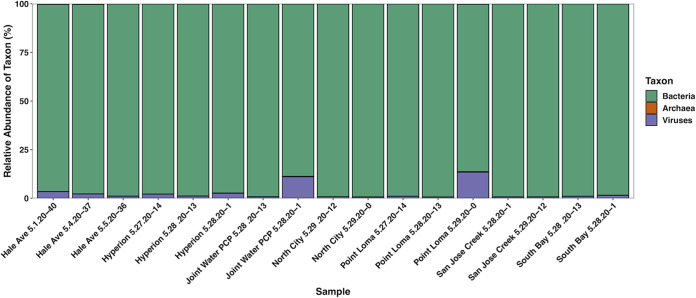
Stacked bar plot indicating the relative abundances of taxa in each sample. The color denotes the taxon, and sample names contain information about each sample, including facility name, sampling date, and number of days stored at 4°C.

We received 45,415,020 total reads (mean, 2.67 million; range, 2.2 to 2.96 million), an average of 33.8% of which were classified by Kraken 2 (range, 9.1 to 43.3%). Of the classified reads, an average of 97.2% were bacterial (range, 86.1 to 99.2%), 0.08% were archaeal (range, 0.02 to 0.26%), and 2.6% were viral (range, 0.65 to 13.4%) (Fig. 1). We found viruses commonly present in wastewater, including CrAssphage (7) and *Pepper mild mottle virus* (6) and, in low abundance, norovirus (13). As we sequenced several bacteriophages with DNA-based genomes, there was likely DNA contamination in our RNA extractions. Interestingly, we detected very few reads of viruses in the family *Coronaviridae*, possibly due to low sequencing depth, storage time, or extraction method (4).

### Data availability.

The raw metagenomic sequence data are available in the NCBI Sequence Read Archive under BioProject number PRJNA649747.
